# Resveratrol protects leukemic cells against cytotoxicity induced by proteasome inhibitors via induction of FOXO1 and p27^Kip1^

**DOI:** 10.1186/1471-2407-11-99

**Published:** 2011-03-19

**Authors:** Xiao-Fang Niu, Bao-Qin Liu, Zhen-Xian Du, Yan-Yan Gao, Chao Li, Ning Li, Yifu Guan, Hua-Qin Wang

**Affiliations:** 1 Department of Biochemistry & Molecular Biology, China Medical University, Shenyang 110001, PR China; 2 Key Laboratory of Cell Biology, Ministry of Public Health, and Key Laboratory of Medical Cell Biology, Ministry of Education, China Medical University, Shenyang 110001, PR China; 3 Department of Endocrinology and Metabolism, the 1stAffiliated Hospital, China Medical University, Shenyang 110001, PR China

## Abstract

**Background:**

It was reported recently that resveratrol could sensitize a number of cancer cells to the antitumoral effects of some conventional chemotherapy drugs. The current study was designed to investigate whether resveratrol could sensitize leukemic cells to proteasome inhibitors.

**Methods:**

Leukemic cells were treated with MG132 alone or in combination with resveratrol. Cell viability was investigated using MTT assay, and induction of apoptosis and cell cycle distribution was measured using flow cytometry. Western blot and real-time RT-PCR were used to investigate the expression of FOXO1 and p27^Kip1^. CHIP was performed to investigate the binding of FOXO1 to the p27 ^Kip1 ^promoter.

**Results:**

Resveratrol strongly reduced cytotoxic activities of proteasome inhibitors against leukemic cells. MG132 in combination with resveratrol caused cell cycle blockade at G1/S transition via p27^Kip1 ^accumulation. Knockdown of p27^Kip1 ^using siRNA dramatically attenuated the protective effects of resveratrol on cytotoxic actions of proteasome inhibitors against leukemic cells. Resveratrol induced FOXO1 expression at the transcriptional level, while MG132 increased nuclear distribution of FOXO1. MG132 in combination with resveratrol caused synergistic induction of p27^Kip1 ^through increased recruitment of FOXO1 on the p27^Kip1 ^promoter.

**Conclusions:**

Resveratrol may have the potential to negate the cytotoxic effects of proteasome inhibitors via regulation of FOXO1 transcriptional activity and accumulation of p27^Kip1^.

## Background

The ubiquitin proteasome system (UPS) is the major proteolytic system encountered in the cytoplasm and nucleus of virtually all nucleated eukaryotic cells[1]. Tight regulation of UPS-mediated proteolysis is maintained to control half-lives of proteins involved in cell cycle regulation, transcriptional control, antigen processing, angiogenesis, and removal of incorrectly folded or damaged proteins[2]. It has become evident that proteasomal function is essential for cell survival and that inhibition of proteasomal activity is a powerful means to induce cytotoxicity in many cancer cells derived from various histology[3,4].

Resveratrol, a naturally occurring polyphenolic compound, is enriched in a variety of food sources, such as grapes, peanuts and red wine. A number of previous studies have reported that resveratrol can inhibit the growth of human cancer cells when it is present alone at rather high concentrations (usually >50 uM) [5-8]. In addition, it has been reported when it is used in combination with other anticancer drugs, resveratrol can avoid some of the debilitating side effects and sensitize a number of cancer cell lines to the anticancer actions of some other conventional chemotherapy drugs such as TNFα, paclitaxel, et al., as well as radiotherapy [5-7,9-13]. Accumulating data support that proteasome inhibitors have the potential to reduce the viability of proliferating cells, while nonproliferating, quiescent cells, in short-term experiments at least, are remarkably protected against apoptosis induced by proteasome inhibitors[14,15]. One common feature of quiescent cells is the upregulation of p27^Kip1^, a ubiquitous cyclin dependent kinase inhibitor (CKI), which leads to G1/S arrest and appears to be a general property of cells that switch to a nonproliferative phenotype[16,17]. In addition, it has been reported that p27^Kip1^-mediated cell cycle arrest at G1/S transition is required for protection against proteasome inhibitors[18].

In the current study, we have found that resveratrol dramatically protects leukemic cells from cytotoxic actions of proteasome inhibitors via p27^Kip1^-mediated G1/S cell cycle arrest. In addition, we have demonstrated that synergistic induction of p27^Kip1 ^via FOXO1 by MG132 in combination with resveratrol is, at least partly, responsible for the protective effects of resveratrol. In light of the recent interest in the resveratrol for its possible use in combination chemotherapy regimens and widespread use of resveratrol among cancer patients, this study calls for more caution for leukemia patients using resveratrol as a dietary adjuvant during treatment with proteasome inhibitors.

## Methods

### Culture of multiple leukemic cell lines

K562, U937, NB4, Daudi and Raji cell lines were maintained in RPMI1640 medium (Sigma-Aldrich, Saint Louis, MO) supplemented with 10% fetal bovine serum (FBS, Sigma-Aldrich, Saint Louis, MO).

### Chemicals

MG132, epoxomycin, PSI and lactacystin were purchased from Calbiochem. 0.02% DMSO was used as vehicle control.

### Cell viability assays

For cell viability assays, cells were plated in 96-well dishes (1 × 10^4 ^cells per well) and treated with different effectors for 24 h. Cell viability was assessed using the 3-(4,5-dimethylthiazol-2-thiazolyl)-2,5-diphenyl tetrazolium bromide (MTT) assay (Chemicon, Bedford, MA) according to the manufacturer's instruction.

### Detection of apoptotic cells

For cell death assays, cells were washed twice in phosphate-buffered saline and then stained with Annexin V-FITC (Biovision, Mountainview, CA) and propidium iodide (PI, Sigma-Aldrich) according to the manufacturer's instructions. After staining with annexin V-FITC and PI, samples were analyzed by fluorescence-activated cell scanner (FACScan) flow cytometer (Becton Dickinson, Franklin Lakes, NJ).

### Analysis of the cell cycle by flow cytometry

Cells were exposed to different concentrations of resveratrol for 24 h. The cells were fixed in 70% ethanol and stained with 50 μg/ml of propidium iodide (PI). The fluorescence was measured using the Becton Dickinson FACScan (Bedford, MA). Distribution of cells in distinct cell cycle phase was determined using ModFIT cell cycle analysis software.

### Western blot analysis

Cells were lysed in lysis buffer (20 mM Tris-HCl, 150 mM NaCl, 2 mM EDTA, 1% Triton-X100 and protease inhibitor cocktail (Sigma-Aldrich, Saint Louis, MO). Cell extract protein amounts were quantified using the BCA protein assay kit. Equivalent amounts of protein (25 μg) were separated using 12% SDS-PAGE and transferred to PVDF membrane (Millipore Corporation, Billerica, MA).

### Preparation of cytoplasmic and nuclear extract

After treatment, cells were lysed in buffer A (containing 10 mM HEPES, pH 7.9, 1.5 mM MgCl_2_, 10 mM KCl, 0.5 mM DTT, 1% Nonidet P-40 and protease inhibitor cocktail) and centrifuged at 12,000 g for 10 min at 4°C. The supernatant was collected and used as the cytoplasmic extracts. The nuclei pellet was resuspended in buffer B (20 mM HEPES, pH7.9, containing 1.5 mM MgCl_2_, 450 mM NaCl, 25% glycerol, 0.2 mM EDTA, 0.5 mM DTT and protease inhibitor cocktail) and agitated fro 60 min at 4°C, and the nuclear debris was spun down at 20,000 g for 15 min. The supernatant (nuclear extract) was collected. Antibodies against Histone H2B and LDH were used as loading controls for nuclear and cytosolic proteins, respectively.

### Chromosomal immunoprecipitation (ChIP) assay

ChIP assays were performed using a kit from Upstate Biotechonology Inc. (Lake Placid, NY) according to the supplied protocol. In brief, cells were exposed to different treatment and fixed with 1% formaldehyde in PBS to cross-link chromatin. Cell lysates were prepared and sonicated on ice to break chromatin DNA to an average length of 400 bp. After a preclearing step, immunoprecipitation was carried out at 4°C overnight with anti-FOXO1 antibody or normal goat IgG (negative control antibody). Immune complexes were collected with salmon sperm DNA saturated protein A-agarose beads. After extensive washing the immunoprecipitated complexes were eluted with 0.1 M NaHCO3 and 1% SDS, and then protein-DNA cross-links were reversed by incubating at 65°C for 5 hours. DNA was purified using proteinase K digestion, phenol: chloroform extraction and ethanol precipitation. Real-time PCR was performed using primers specific for the p27^Kip1 ^sequence between -237 and +15 (forward: 5'-AGGTTTGTTGGCAGCAGTACC-3' and reverse: 5'-AGGCTGACGAAGAAGAAAATG-3') to generate a 252 bp amplification product containing the FOXO response element[19,20]. A standard curve was prepared using serial dilutions of PCR products using genomic DNA as template. The amount of p27^Kip1 ^promoter fragment that was present in the immunoprecipitation and input fractions was calculated from the standard curve. The input represents 1% of the material used in the immunoprecipitation assay. The results were expressed as the immunoprecipitation/input ratios of the PCR products were used for comparison.

### Small interfering RNA

The siRNA sequences used here were as follows: siRNA against p27^Kip1 ^(sip27^Kip1^), GGAGCAAUGCGCAGGAAUAUU; siRNA against FOXO1 (siFOXO1), CCCUGUAACUGACAGACCAAAU. The scramble nonsense siRNA (scramble; CCGUAUCGUAAGCAGUACU) that has no homology to any known genes was used as control. The cells were transfected using FuGENE 6 according to the manufacturer's instruction.

### Statistics

The statistical significance of the difference was analyzed by ANOVA and post hoc Dunnett's test. Statistical significance was defined as p < 0.05. All experiments were repeated three times, and the results are presented as mean ± standard deviation (SD) of the three repeated experiments performed in triplicate.

## Results

### Resveratrol suppresses the cytotoxic effects of proteasome inhibitors in K562 leukemic cells

**Figure 1:**
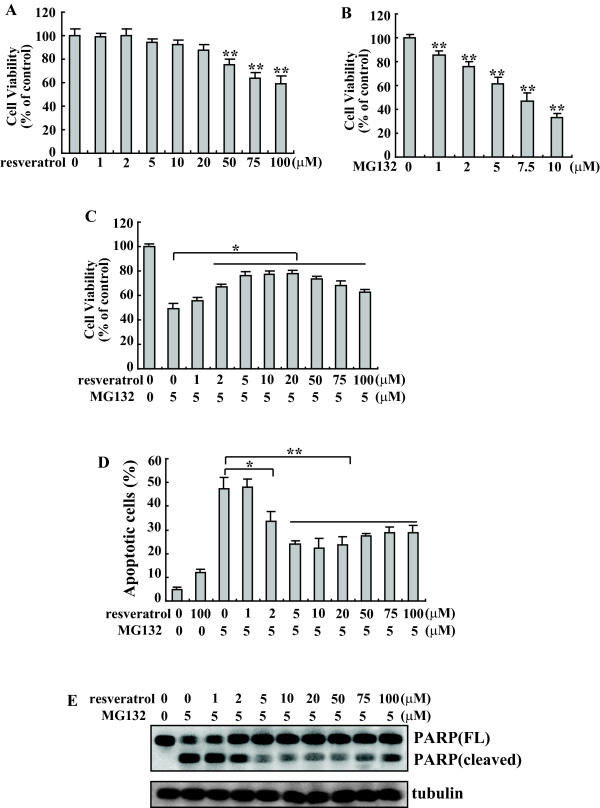
**Resveratrol blocks the cytotoxic effects of MG132 in K562 cells**. A-B, Cell viability of K562 cells was determined using MTT assay after treatment with various concentrations of resveratrol (A) and MG132 (B) for 24 h, respectively. C, The percentage of viable cells was determined using MTT assay after treatment with 5 μM MG132 and various concentrations of resveratrol for 24 h. D, Cells were treated with 5 μM MG132 and various concentration of resveratrol for 24 h, apoptotic cells were analyzed. A-D, The results are presented as mean of three independent experiments performed in triplicate, and error bars represent standard deviation. E, K562 cells were treated with 5 μM MG132 and various concentration of resveratrol for 24 h, and Western blot analysis was performed. Representative blot from three independent experiments with similar results was shown. *, *P *< 0.05; **, *P *< 0.01.

Cell viability of K562 cells was decreased upon treatment with resveratrol when used higher concentration than 50 μM, while 1-20 μM of resveratrol had no obvious effects on K562 cell viability within 24 h (Figure 1A). Viability of K562 cells treated with MG132 demonstrated a dose-dependent decrease within 24 h (Figure 1B). MTT assay of K562 cells demonstrated that resveratrol significantly prevented the cytotoxicity induced by MG132 in a dose-dependent manner (Figure 1C). As low as 2 μM of resveratrol demonstrated obvious protective effect, with the maximal protective effect observed at the concentrations among 5 to 20 μM (Figure 1C). Interestingly, even under conditions where resveratrol was obviously toxic at the concentrations of 50 to 100 μM, it was still able to antagonize the cytotoxic effect of MG132 (Figure 1C). MG132-induced apoptosis was further determined by flow-cytometric analysis of K562 cells labeled with propidium iodide (PI) and annexin V. Consistent with our previous report [21], resveratrol alone caused only about 12% of apoptotic cells within 24 h when applied even at concentrations of 100 μM (Figure 1D). The increase in apoptotic cells induced by 5 μM MG132 alone was markedly abrogated by the addition of resveratrol (Figure 1D). Furthermore, the addition of resveratrol significantly inhibited MG132-induced PARP cleavage to the characteristic apoptotic 89 kDa fragment (Figure 1E). These results confirmed the protective effect of resveratrol on MG132-induced apoptosis in K562 cells.

### Resveratrol acts as a survival factor in human leukemic cells against proteasome inhibition

**Figure 2:**
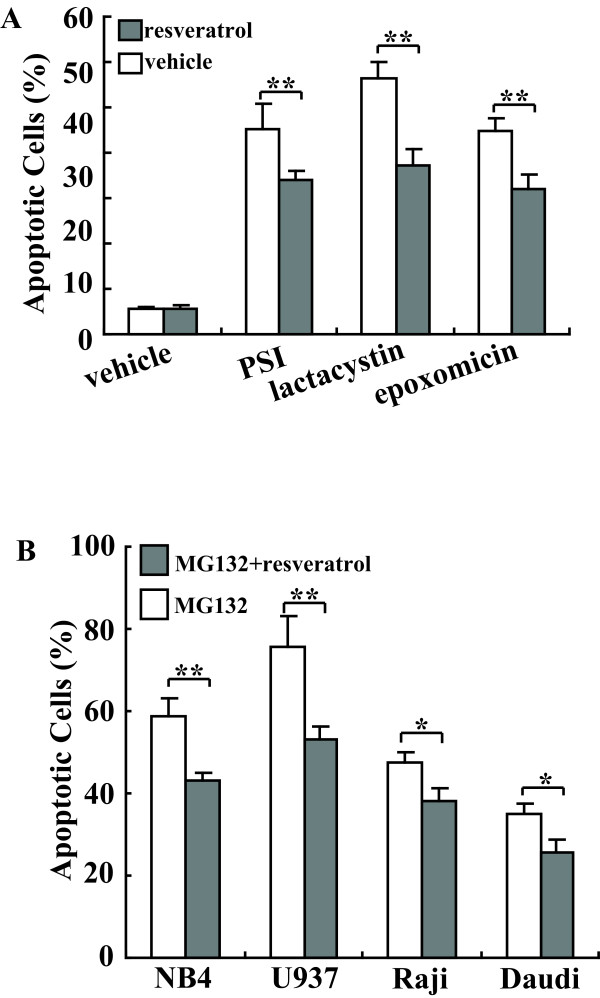
**Protective roles of resveratrol against proteasome inhibition in a panel of leukemic cells**. A, K562 cells were treated with 5 nM PSI, 10 μM lactacystin or 50 nM epoxomicin alone or combination with 5 μM resveratrol for 24 h. Apoptotic cells were analyzed using Annexin-FITC/PI double staining followed by FACS analysis. B, A panel of leukemic cells was treated with 5 μM MG132 alone or combination with 5 μM resveratrol for 24 h, and apoptotic cells were analyzed using Annexin-FITC/PI double staining followed by FACS analysis. The results are presented as mean of three independent experiments performed in triplicate, and error bars represent standard deviation. *, *P *< 0.05; **, *P *< 0.01.

We then further determined whether the protective effect of resveratrol was a general phenomenon against proteasome inhibitors. Since 5-20 μM of resveratrol demonstrated the maximal protective effect on MG132-mediated cytotoxicity of K562 cells (Figure 1C-E), we used 5 μM of resveratrol in the following experiments. Three structurally different proteasome inhibitors, namely PSI (5 nM), lactacystin (10 μM) as well as epoxomicin (50 nM) were able to induce up to 40-60% apoptosis of K562 cells within 24 h of incubation (Figure 2A). A marked protection of K562 against the cytotoxic effects of all three proteasome inhibitors could be observed when used in combination with resveratrol (Figure 2A). To further test the potential effect of resveratrol as a survival factor, we extended our studies to various human leukemic cell lines. Resveratrol also exerted a protective effect against cytotoxicity induced by MG132 in other four human leukemic cell lines, NB4, U937, Daudi and Raji (Figure 2B). These findings indicated that resveratrol antagonized the cytotoxic actions of proteasome inhibitors in a variety of human leukemic cell lines.

### Involvement of p27^Kip1^-mediated G1/S arrest in the protective effects of resveratrol against cytotoxicity induced by MG132

**Figure 3:**
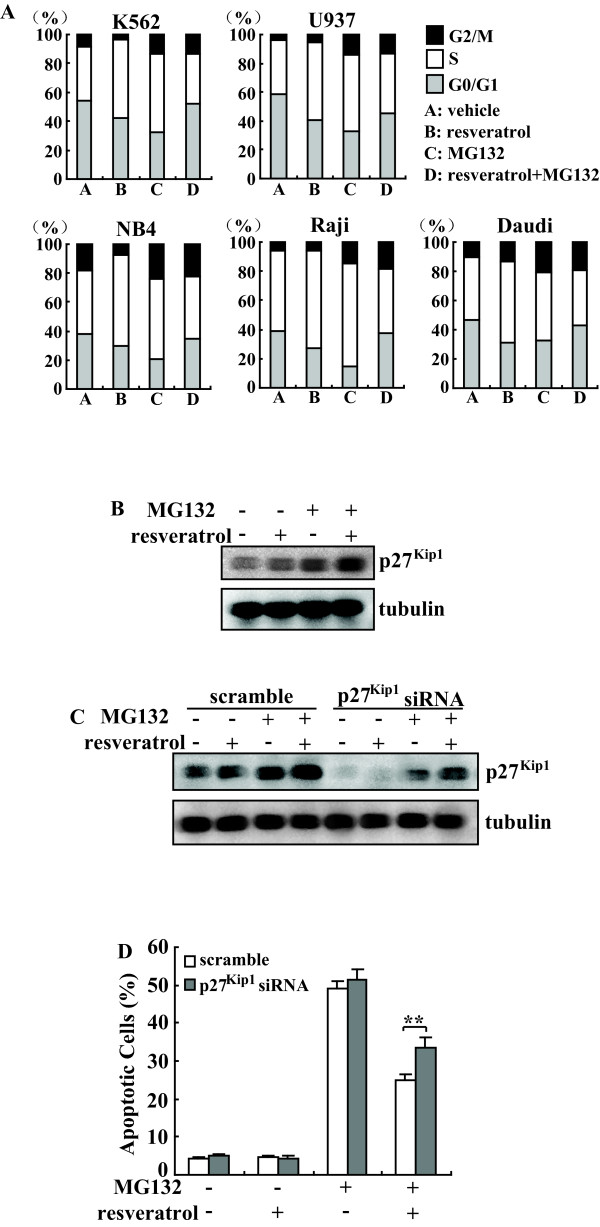
**Involvement of p27^Kip1^-mediated G1/S arrest in the protective effects of resveratrol against MG132-mediated cell death**. A, The panel of leukemic cells was treated with 5 μM resveratrol or 5 μM MG132 alone or combination for 24 h and cell cycle distribution was measured using FACS analysis. Representative graph from three independent experiments with similar results was shown. B, K562 cells were treated as A, and Western blot analysis was performed. Representative blot from three independent experiments with similar results was shown. C, K562 cells were transfected with scramble or p27^Kip1 ^siRNA for 24 h, then treated with 5 μM resveratrol or 5 μM MG132 alone or combination for another 24 h. p27^Kip1 ^expression levels were investigated using Western blot analysis. Representative blot from three independent experiments with similar results was shown. D, Cells were treated as C, and apoptotic cells were measured using Annexin-FITC/PI double staining followed by FACS analysis. The results are presented as mean of three independent experiments performed in triplicate, and error bars represent standard deviation. *, *P *< 0.05; **, *P *< 0.01.

5 μM Resveratrol primarily increased cells in the S phase, whereas 5 μM MG132 predominantly caused an increase of cells in the S and G2/M phase (Figure 3A), as determined by propidium iodide (PI) staining and FACS analysis of cells incubated with resveratrol or MG132 for 24 h. MG132 in combination with resveratrol significantly decreased the population of cells in the S phase, but increased the population in the G1 phase when compared with MG132 alone (Figure 3A). Since rapidly proliferating cells were much sensitive to proteasome inhibitor-mediated apoptosis versus their quiescent counterparts[15,22-26], furthermore, blockade at G1/S transition appears to be a general property of cells that switch to a nonproliferative phenotype[16,17], these results suggested that antagonism of resveratrol against MG132 might be the result of blocking cell cycle progression at the G1/S transition and thus preventing the cell from proliferation.

The cyclin dependent kinase (CDK) inhibitor p27^Kip1 ^is an important regulator of cell cycle progression controlling the transition from G1 to S-phase [27], in addition, it has also been reported that p27^Kip1^-mediated cell cycle arrest at the G1/S transition is required to confer protection for K562 cells against proteasome inhibitors[18]. Therefore, we investigated whether p27^Kip1 ^also contributed to the protective effect of resveratrol against proteasome inhibitors-induced cytotoxicity in leukemic cells. Resveratrol or MG132 alone increased p27^Kip1^, combinational treatment with MG132 and resveratrol further enhanced the expression of p27^Kip1 ^(Figure 3B). To clarify the potential involvement of p27^Kip1 ^accumulation in protective effects of resveratrol, p27^Kip1 ^expression was knocked down using the p27^Kip1 ^specific siRNA. Accumulation of p27^Kip1 ^mediated by MG132, resveratrol, or their combination was decreased in p27^Kip1^-knockdown cells (Figure 3C). Knockdown of p27^Kip1 ^provided marginal protection against MG132 alone-mediated apoptosis (Figure 3D). Importantly, the protective effect of resveratrol against MG132 was significantly weakened by p27^Kip1 ^Knockdown (Figure 3D). These results indicated that resveratrol-induced p27^Kip1^, at least partially contributed to the resveratrol-mediated attenuation of the apoptotic effects of MG132.

### Involvement of FOXO1 in upregulation of p27^Kip1 ^induced by resveratrol and MG132

**Figure 4:**
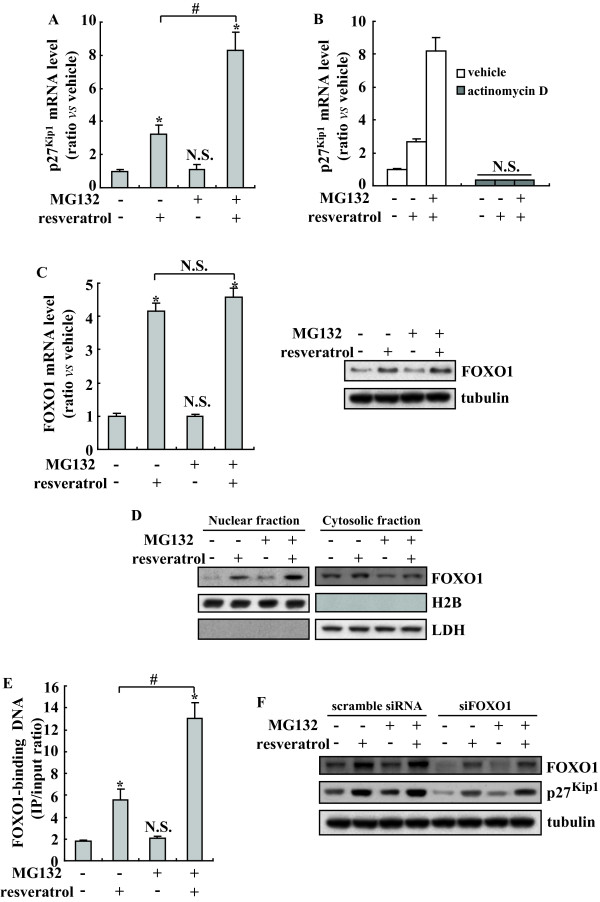
**Involvement of FOXO1 in induction of p27^Kip1 ^mediated by resveratrol and MG132**. A, K562 cells were treated with 5 μM resveratrol or 5 μM MG132 alone or combination for 8 h and real-time RT-PCR was performed. The results are presented as mean of three independent experiments performed in triplicate, and error bars represent standard deviation. B, K562 cells were pretreated with vehicle or actinomycin D for 1 h, then treated with resveratrol alone or combination with MG132 for additional 8 h, then real-time RT-PCR was performed. The results are presented as mean of three independent experiments performed in triplicate, and error bars represent standard deviation. C, K562 cells were treated with 5 μM resveratrol or 5 μM MG132 alone or combination, FOXO1 mRNA and protein levels were investigated using real-time RT-PCR and Western blot, respectively. The results of real-time RT-PCR are presented as mean of three independent experiments performed in triplicate, and error bars represent standard deviation. Representative blot from three independent experiments with similar results was shown. D, K562 cells were treated as C, Western blot was performed on cytosolic and nuclear proteins, respectively. Representative blot from three independent experiments with similar results was shown. E, K562 cells were exposed to MG132 or resveratrol alone or combination for 8 h. Cross-linked chromatin was extracted and immunoprecipitated with an anti-FOXO1 antibody. Immunoprecipitated DNA was amplified by real-time RCR. The results are presented as mean of three independent experiments performed in triplicate, and error bars represent standard deviation. F, K562 cells were transfected with scramble or siRNA against FOXO1 (siFOXO1) for 48 h, then treated with MG132 or resveratrol alone or combination for additional 24 h, and Western blot was performed. Representative blot from three independent experiments with similar results was shown. *, *P *< 0.01 *vs *control; #, *P *< 0.01.

We then examined the mechanism underlying upregulation of p27^Kip1 ^by resveratrol or MG132. Real-time PCR showed that resveratrol alone increased p27^Kip1 ^mRNA, whereas MG132 alone had no obvious effect on p27^Kip1 ^mRNA expression (Figure 4A). Co-administration of resveratrol with MG132 significantly augmented p27^Kip1 ^mRNA when compared with resveratrol alone (Figure 4A). To determine whether *de novo *RNA synthesis is required for the elevation of p27^Kip1^, actinomycin D, an inhibitor of RNA synthesis was pre-administrated before treatment with resveratrol alone or in combination with MG132. Actinomycin D completely blocked upregulation of p27^Kip1 ^mRNA by resveratrol alone or in combination with MG132 (Figure 4B).

Resveratrol enhances the recruitment of transcription factor forkhead box class O transcription factor (FOXO)1 to the FOXO-binding element [28,29]. In addition, FOXO1 has been shown to trans-activate p27^Kip1 ^expression [30-32]. To verify whether there is a correlation between resveratrol-mediated induction of p27^Kip1 ^and the activation of FOXO1 pathway, we tested the expression of FOXO1 by real-time RT-PCR and Western blot. FOXO1 expression was notably increased with resveratrol treatment (either in resveratrol alone or in combination with MG132 group) compared with the groups treated with vehicle or MG132 alone, whereas there was no obvious difference between resveratrol alone and in combination with MG132 (Figure 4C). Investigation of the cellular distribution of FOXO1 demonstrated that FOXO1 localized primarily to the cytoplasm in the vehicle-treated cells, nuclear FOXO1 was significantly increased in cells treated with resveratrol alone or in combination with MG132 (Figure 4D). With some lesser extent, MG132 alone also increased nuclear localization of FOXO1 (Figure 4D). To see whether the different distribution of FOXO1 is ascribed to the synergistic upregulation of p27^Kip1 ^by MG132 in combination with resveratrol via recruitment of FOXO1 to the FOXO-binding site of p27^Kip1 ^promoter, we then performed ChIP analysis and found that binding of FOXO1 to the p27^Kip1 ^promoter was enhanced in cells co-treated with resveratrol and MG132, compared with those treated with resveratrol alone (Figure 4E). To confirm whether FOXO1 is responsible for upregulation of p27^Kip1^, we used specific siRNA against FOXO1 to verify its effect on the expression of p27^Kip1^. Specific siRNA against FOXO1 effectively suppressed upregulation of FOXO1 induced by resveratrol treatment (Figure 4F). Importantly, concomitant with FOXO1 reduction, resveratrol-induced p27^Kip1 ^expression was suppressed in cells transfected with siRNA against FOXO1 (Figure 4F).

## Discussion

Single agent of proteasome inhibitor resulted in significant responses in leukemic cells and the combination of proteasome inhibitors and other chemotherapeutic drugs enhanced its antitumoral efficacy [3,33-37]. Initially, the experiments were planned to test whether resveratrol could sensitized K562 cells to the anticancer actions of proteasome inhibitors. To our surprise, resveratrol did not promote, but rather attenuated the apoptotic effects of MG132 in cultured K562 cells. We further extended our investigation using a panel of leukemic cells and found that resveratrol also attenuated the cytotoxic actions of MG132 in NB4, U937, Raji and Daudi cells. Furthermore, resveratrol also compromised the apoptotic effects of other three structurally different proteasome inhibitors, PSI, epoxomicin and lactacystin. This was consistent with the previous study that resveratrol exerted its protective effects against proteasome inhibitor-induced cellular damages in human skeletal myotubes [38]. Consistent with our previous report[21], in the current study, we found that resveratrol *per se *did not cause obvious apoptosis when less than 100 μM concentration was used within 24 h. Chakraborty PK et al. reported that treatment with 40 μM resveratrol for 48 h induced apoptosis of K562 cells [39]. The different effects of resveratrol on apoptosis of K562 cells might be ascribed to different period of exposure. Alternatively, Chakraborty PK et al. used subG1 fractions represented as apoptotic cells [39], while in the current study, we used Annexin V/PI double staining followed by flow cytometry to detect apoptotic cells. The different methods used in these studies might contribute to the different apoptotic actions of resveratrol. The higher cytoprotective effect of resveratrol on cytotoxic actions of proteasome inhibitors was observed when it was used at 5-20 μM concentration. We observed that 50-100 μM resveratrol was slightly cytotoxic for K562 cells, which could explain why this concentration exerted a lower cytoprotective action compared with 20 μM resveratrol. Even this, when cells were concurrently incubated with 100 μM of resveratrol, the apoptosis observed after exposure to MG132 was significantly lower than the one observed in the cells exposed to MG132 alone, indicating that even when 100 μM resveratrol could induce a certain degree of cytotoxicity in these cells, at the same time exerted a cytoprotective action against cytotoxicity-mediated by proteasome inhibition. Resveratrol was reported to be abundant in grapes, blueberries and peanuts. In grapes, its highest concentration was in the skin (50-100 μg per gram), thereby making red wines (but not white wines) the richest dietary source [40]. In plasma, it bound with lipoproteins and albumin which facilitated its carrier-mediated cellular uptake [41]. In experimental animals, resveratrol was rapidly metabolized by the liver and its plasma half-life remained quite low [42], however, in human, about 70% of orally administered resveratrol (25 mg) was absorbed with a peak plasma level of ~2 μM and a half-life of ~10 h [43]. In the current study, we found that 5 μM of resveratrol could antagonize the cytotoxic effects of proteasome inhibitors. Therefore, concurrent intake of resveratrol products should be discreet.

Arrest at G1/S transition appeared to be a general property of cells that switched to a nonproliferative phenotype [16,17,44]. Compared with nonproliferating, quiescent cells, proliferating cells were much more sensitive to cytotoxicity induced by proteasome inhibitors [14,15]. In the current study, we found that combination of resveratrol and MG132 significantly increased proportion of cells in G1 fraction, therefore, protective effects of resveratrol against proteasome inhibition might be the result of blocking cell cycle progression at the G1/S transition and thus preventing the cells from proliferation.

Proteasome inhibitor-induced apoptosis generally was accompanied by the accumulation of p27^Kip1^, a universal CDK-cyclin inhibitor responsible for cell cycle arrest at G1/S transition [45]. A rather broad spectrum of effects were ascribed to elevated levels of p27^Kip1 ^protein ranging from proapoptotic functions in various systems to survival-promoting properties in others. Conflicting observations were also reported regarding the role of p27^Kip1 ^in apoptosis induced by proteasome inhibitors. As overexpression of p27^Kip1 ^in various tumor cell lines was sufficient to induce apoptosis in various cancer cell lines [46,47], it had therefore been deduced that cytotoxicity induced by proteasome inhibitors could be due to the uncoordinated upregulation of p27^Kip1 ^[45,48,49]. These pro-apoptotic properties were also consistent with the notion that p27^Kip1 ^exerted the task of a tumor suppressor gene. In contrast to these observations, the cytotoxic effects of proteasome inhibitors in general appeared to be selective for proliferating cells, but quiescent cells generally with high levels of p27^Kip1 ^in nucleus seemed to be protected [14,15]. For example, primary endothelial cells which became contact inhibited upon reaching confluence displayed a remarkable degree of resistance against apoptosis induced by proteasome inhibitors in the presence of increased steady state levels of p27^Kip1^, when compared with their proliferating counterparts [15]. Similar observations were also observed in different cancer cell lines engineered to overexpress p27^Kip1 ^[50-52]. Likewise, inducible overexpression of p27^Kip1 ^protected K562 cells against induction of apoptosis by proteasome inhibitors [18]. Since proliferation and differentiation were usually mutually exclusive, it was not surprised that cell cycle arrest at G1/S transition and p27^Kip1 ^was also involved in the differentiation of erythroid precursors [53,54]. Thus, induction of cell differentiation via accumulation of p27^Kip1 ^and G1/S arrest might also contribute to the protective roles of resveratrol against proteasome inhibition-mediated cytotoxicity.

A major consequence of the anti-apoptotic properties of p27^Kip1 ^appeared that high levels of p27^Kip1 ^in tumor cells might not be always good news for cancer patients: high levels of active p27^Kip1 ^within tumor cells might indicate that although less aggressive and more slowly growing, this tumor might be more difficult to be attacked by treatment with proteasome inhibitors or other chemotherapeutic drugs.

In conclusion, the present study demonstrated that resveratrol had the potential to negate the therapeutic efficacy of proteasome inhibitors in leukemic cells and suggested that intake of resveratrol-related products might be contraindicated for patients undergoing treatment with proteasome inhibitors. Considering the widespread use of resveratrol among cancer patients, further investigations should be necessary to elucidate the *in vivo *significance of these findings, which in turn might inform the need for dietary advice on the consumption of resveratrol during chemotherapy with proteasome inhibitors.

## Conclusions

Resveratrol may have the potential to negate the cytotoxic effects of proteasome inhibitors via regulation of FOXO1 transcriptional activity and accumulation of p27^Kip1^. Further investigations should be performed to elucidate the *in vivo *significance of these findings, which in turn might inform patients undergoing the chemotherapy with proteasome inhibitors to avoid intake of resveratrol-related products.

## Competing interests

The authors declare that they have no competing interests.

## Authors' contributions

XFN carried out the cell culture and molecular studies, and participated in the data analysis. BQL carried out ChIP, nuclear fractionation and flow cytometry. ZXD participated in real-time PCR and cell culture. YYG participated in flow cytometry and MTT assay. CL participated in cell culture and flow cytometry.NL participated in the DNA cloning and Western blot analysis. YG participated in manuscript proofreading. HQW conceived of the study, and participated in manuscript drafting and coordinate. All authors read and approved the final manuscript.

## Pre-publication history

The pre-publication history for this paper can be accessed here:

http://www.biomedcentral.com/1471-2407/11/99/prepub

## References

[B1] Hershko A Ciechanover A The ubiquitin system Annu Rev Biochem 1998 67 425 479 10.1146/annurev.biochem.67.1.425 9759494

[B2] Baumeister W Walz J Zuhl F Seemuller E The proteasome: paradigm of a self-compartmentalizing protease Cell 1998 92 3 367 380 10.1016/S0092-8674(00)80929-0 9476896

[B3] Jagani Z Song K Kutok JL Dewar MR Melet A Santos T Grassian A Ghaffari S Wu C Yeckes-Rodin H Proteasome inhibition causes regression of leukemia and abrogates BCR-ABL-induced evasion of apoptosis in part through regulation of forkhead tumor suppressors Cancer Res 2009 69 16 6546 6555 10.1158/0008-5472.CAN-09-0605 19654305 PMC2763358

[B4] Deleu S Lemaire M Arts J Menu E Van Valckenborgh E Vande Broek I De Raeve H Coulton L Van Camp B Croucher P Bortezomib alone or in combination with the histone deacetylase inhibitor JNJ-26481585: effect on myeloma bone disease in the 5T2 MM murine model of myeloma Cancer Res 2009 69 13 5307 5311 10.1158/0008-5472.CAN-08-4472 19531653

[B5] Fulda S Debatin KM Sensitization for tumor necrosis factor-related apoptosis-inducing ligand-induced apoptosis by the chemopreventive agent resveratrol Cancer Res 2004 64 1 337 346 10.1158/0008-5472.CAN-03-1656 14729643

[B6] Gill C Walsh SE Morrissey C Fitzpatrick JM Watson RW Resveratrol sensitizes androgen independent prostate cancer cells to death-receptor mediated apoptosis through multiple mechanisms Prostate 2007 67 15 1641 1653 10.1002/pros.20653 17823925

[B7] Fulda S Debatin KM Sensitization for anticancer drug-induced apoptosis by the chemopreventive agent resveratrol Oncogene 2004 23 40 6702 6711 10.1038/sj.onc.1207630 15273734

[B8] Zhou R Fukui M Choi HJ Zhu BT Induction of a reversible, non-cytotoxic S-phase delay by resveratrol: implications for a mechanism of lifespan prolongation and cancer protection Br J Pharmacol 2009 158 2 462 474 10.1111/j.1476-5381.2009.00268.x 19563536 PMC2757685

[B9] Kubota T Uemura Y Kobayashi M Taguchi H Combined effects of resveratrol and paclitaxel on lung cancer cells Anticancer Res 2003 23 5A 4039 4046 14666716

[B10] Jazirehi AR Bonavida B Resveratrol modifies the expression of apoptotic regulatory proteins and sensitizes non-Hodgkin's lymphoma and multiple myeloma cell lines to paclitaxel-induced apoptosis Mol Cancer Ther 2004 3 1 71 84 10.4161/cbt.3.1.683 14749477

[B11] Ivanov VN Partridge MA Johnson GE Huang SX Zhou H Hei TK Resveratrol sensitizes melanomas to TRAIL through modulation of antiapoptotic gene expression Exp Cell Res 2008 314 5 1163 1176 10.1016/j.yexcr.2007.12.012 18222423 PMC3738302

[B12] Scarlatti F Sala G Ricci C Maioli C Milani F Minella M Botturi M Ghidoni R Resveratrol sensitization of DU145 prostate cancer cells to ionizing radiation is associated to ceramide increase Cancer Lett 2007 253 1 124 130 10.1016/j.canlet.2007.01.014 17321671

[B13] Bhardwaj A Sethi G Vadhan-Raj S Bueso-Ramos C Takada Y Gaur U Nair AS Shishodia S Aggarwal BB Resveratrol inhibits proliferation, induces apoptosis, and overcomes chemoresistance through down-regulation of STAT3 and nuclear factor-kappaB-regulated antiapoptotic and cell survival gene products in human multiple myeloma cells Blood 2007 109 6 2293 2302 10.1182/blood-2006-02-003988 17164350

[B14] Drexler HC Activation of the cell death program by inhibition of proteasome function Proc Natl Acad Sci USA 1997 94 3 855 860 10.1073/pnas.94.3.855 9023346 PMC19603

[B15] Drexler HC Risau W Konerding MA Inhibition of proteasome function induces programmed cell death in proliferating endothelial cells Faseb J 2000 14 1 65 77 10627281 10.1096/fasebj.14.1.65

[B16] Hirano M Hirano K Nishimura J Kanaide H Transcriptional up-regulation of p27(Kip1) during contact-induced growth arrest in vascular endothelial cells Exp Cell Res 2001 271 2 356 367 10.1006/excr.2001.5384 11716548

[B17] Polyak K Kato JY Solomon MJ Sherr CJ Massague J Roberts JM Koff A p27Kip1, a cyclin-Cdk inhibitor, links transforming growth factor-beta and contact inhibition to cell cycle arrest Genes Dev 1994 8 1 9 22 10.1101/gad.8.1.9 8288131

[B18] Drexler HC Pebler S Inducible p27(Kip1) expression inhibits proliferation of K562 cells and protects against apoptosis induction by proteasome inhibitors Cell Death Differ 2003 10 3 290 301 10.1038/sj.cdd.4401159 12700629

[B19] Lynch RL Konicek BW McNulty AM Hanna KR Lewis JE Neubauer BL Graff JR The progression of LNCaP human prostate cancer cells to androgen independence involves decreased FOXO3a expression and reduced p27KIP1 promoter transactivation Mol Cancer Res 2005 3 3 163 169 10.1158/1541-7786.MCR-04-0163 15798096

[B20] Tang ED Nunez G Barr FG Guan KL Negative regulation of the forkhead transcription factor FKHR by Akt J Biol Chem 1999 274 24 16741 16746 10.1074/jbc.274.24.16741 10358014

[B21] Liu BQ Gao YY Niu XF Xie JS Meng X Guan Y Wang HQ Implication of unfolded protein response in resveratrol-induced inhibition of K562 cell proliferation Biochem Biophys Res Commun 2010 391 1 778 782 10.1016/j.bbrc.2009.11.137 19944671

[B22] Yin D Zhou H Kumagai T Liu G Ong JM Black KL Koeffler HP Proteasome inhibitor PS-341 causes cell growth arrest and apoptosis in human glioblastoma multiforme (GBM) Oncogene 2005 24 3 344 354 10.1038/sj.onc.1208225 15531918

[B23] Bazzaro M Lee MK Zoso A Stirling WL Santillan A Shih Ie M Roden RB Ubiquitin-proteasome system stress sensitizes ovarian cancer to proteasome inhibitor-induced apoptosis Cancer Res 2006 66 7 3754 3763 10.1158/0008-5472.CAN-05-2321 16585202

[B24] Soligo D Servida F Delia D Fontanella E Lamorte G Caneva L Fumiatti R Lambertenghi Deliliers G The apoptogenic response of human myeloid leukaemia cell lines and of normal and malignant haematopoietic progenitor cells to the proteasome inhibitor PSI Br J Haematol 2001 113 1 126 135 10.1046/j.1365-2141.2001.02683.x 11328292

[B25] Orlowski RZ Eswara JR Lafond-Walker A Grever MR Orlowski M Dang CV Tumor growth inhibition induced in a murine model of human Burkitt's lymphoma by a proteasome inhibitor Cancer Res 1998 58 19 4342 4348 9766662

[B26] Nasr R El-Sabban ME Karam JA Dbaibo G Kfoury Y Arnulf B Lepelletier Y Bex F de The H Hermine O Efficacy and mechanism of action of the proteasome inhibitor PS-341 in T-cell lymphomas and HTLV-I associated adult T-cell leukemia/lymphoma Oncogene 2005 24 3 419 430 10.1038/sj.onc.1208212 15543232

[B27] Agrawal D Hauser P McPherson F Dong F Garcia A Pledger WJ Repression of p27kip1 synthesis by platelet-derived growth factor in BALB/c 3T3 cells Mol Cell Biol 1996 16 8 4327 4336 8754833 10.1128/mcb.16.8.4327 PMC231431

[B28] Srivastava RK Unterman TG Shankar S FOXO transcription factors and VEGF neutralizing antibody enhance antiangiogenic effects of resveratrol Mol Cell Biochem 2010 337 1-2 201 212 10.1007/s11010-009-0300-5 20012470 PMC4153854

[B29] Ganjam GK Dimova EY Unterman TG Kietzmann T FoxO1 and HNF-4 are involved in regulation of hepatic glucokinase gene expression by resveratrol J Biol Chem 2009 284 45 30783 30797 10.1074/jbc.M109.045260 19740748 PMC2781477

[B30] Medema RH Kops GJ Bos JL Burgering BM AFX-like Forkhead transcription factors mediate cell-cycle regulation by Ras and PKB through p27kip1 Nature 2000 404 6779 782 787 10.1038/35008115 10783894

[B31] Dijkers PF Medema RH Pals C Banerji L Thomas NS Lam EW Burgering BM Raaijmakers JA Lammers JW Koenderman L Forkhead transcription factor FKHR-L1 modulates cytokine-dependent transcriptional regulation of p27(KIP1) Mol Cell Biol 2000 20 24 9138 9148 10.1128/MCB.20.24.9138-9148.2000 11094066 PMC102172

[B32] Nakamura N Ramaswamy S Vazquez F Signoretti S Loda M Sellers WR Forkhead transcription factors are critical effectors of cell death and cell cycle arrest downstream of PTEN Mol Cell Biol 2000 20 23 8969 8982 10.1128/MCB.20.23.8969-8982.2000 11073996 PMC86551

[B33] Hu Z Pan XF Wu FQ Ma LY Liu DP Liu Y Feng TT Meng FY Liu XL Jiang QL Synergy between proteasome inhibitors and imatinib mesylate in chronic myeloid leukemia PLoS One 2009 4 7 e6257 10.1371/journal.pone.0006257 19606213 PMC2705802

[B34] Shah JJ Orlowski RZ Proteasome inhibitors in the treatment of multiple myeloma Leukemia 2009 23 11 1964 1979 10.1038/leu.2009.173 19741722 PMC4737506

[B35] Paoluzzi L Scotto L Marchi E Seshan VE O'Connor OA The anti-histaminic cyproheptadine synergizes the antineoplastic activity of bortezomib in mantle cell lymphoma through its effects as a histone deacetylase inhibitor Br J Haematol 2009 146 6 656 659 10.1111/j.1365-2141.2009.07797.x 19604235

[B36] Zhang QL Wang L Zhang YW Jiang XX Yang F Wu WL Janin A Chen Z Shen ZX Chen SJ The proteasome inhibitor bortezomib interacts synergistically with the histone deacetylase inhibitor suberoylanilide hydroxamic acid to induce T-leukemia/lymphoma cells apoptosis Leukemia 2009 23 8 1507 1514 10.1038/leu.2009.41 19282831

[B37] Drexler HC Synergistic apoptosis induction in leukemic cells by the phosphatase inhibitor salubrinal and proteasome inhibitors PLoS One 2009 4 1 e4161 10.1371/journal.pone.0004161 19129918 PMC2613525

[B38] Touzet O Philips A Resveratrol protects against protease inhibitor-induced reactive oxygen species production, reticulum stress and lipid raft perturbation Aids 2010 24 10 1437 1447 10.1097/QAD.0b013e32833a6114 20539089

[B39] Chakraborty PK Mustafi SB Ganguly S Chatterjee M Raha S Resveratrol induces apoptosis in K562 (chronic myelogenous leukemia) cells by targeting a key survival protein, heat shock protein 70 Cancer Sci 2008 99 6 1109 1116 10.1111/j.1349-7006.2008.00809.x 18429957 PMC11159327

[B40] Goswami SK Das DK Resveratrol and chemoprevention Cancer Lett 2009 284 1 1 6 10.1016/j.canlet.2009.01.041 19261378

[B41] Jannin B Menzel M Berlot JP Delmas D Lancon A Latruffe N Transport of resveratrol, a cancer chemopreventive agent, to cellular targets: plasmatic protein binding and cell uptake Biochem Pharmacol 2004 68 6 1113 1118 10.1016/j.bcp.2004.04.028 15313407

[B42] Asensi M Medina I Ortega A Carretero J Bano MC Obrador E Estrela JM Inhibition of cancer growth by resveratrol is related to its low bioavailability Free Radic Biol Med 2002 33 3 387 398 10.1016/S0891-5849(02)00911-5 12126761

[B43] Walle T Hsieh F DeLegge MH Oatis JE Jr Walle UK High absorption but very low bioavailability of oral resveratrol in humans Drug Metab Dispos 2004 32 12 1377 1382 10.1124/dmd.104.000885 15333514

[B44] Li L Zhang G Zhang Y Tan J Huang H Huang B Lu J Sodium butyrate-induced upregulation of p18(INK4C) gene affects K562 cell G (0)/G (1) arrest and differentiation Mol Cell Biochem 2008 319 1-2 9 15 10.1007/s11010-008-9870-x 18642058

[B45] An B Goldfarb RH Siman R Dou QP Novel dipeptidyl proteasome inhibitors overcome Bcl-2 protective function and selectively accumulate the cyclin-dependent kinase inhibitor p27 and induce apoptosis in transformed, but not normal, human fibroblasts Cell Death Differ 1998 5 12 1062 1075 10.1038/sj.cdd.4400436 9894613

[B46] Katayose Y Kim M Rakkar AN Li Z Cowan KH Seth P Promoting apoptosis: a novel activity associated with the cyclin-dependent kinase inhibitor p27 Cancer Res 1997 57 24 5441 5445 9407946

[B47] Wang X Gorospe M Huang Y Holbrook NJ p27Kip1 overexpression causes apoptotic death of mammalian cells Oncogene 1997 15 24 2991 2997 10.1038/sj.onc.1201450 9416843

[B48] Kudo Y Takata T Ogawa I Kaneda T Sato S Takekoshi T Zhao M Miyauchi M Nikai H p27Kip1 accumulation by inhibition of proteasome function induces apoptosis in oral squamous cell carcinoma cells Clin Cancer Res 2000 6 3 916 923 10741716

[B49] Sun J Nam S Lee CS Li B Coppola D Hamilton AD Dou QP Sebti SM CEP1612, a dipeptidyl proteasome inhibitor, induces p21WAF1 and p27KIP1 expression and apoptosis and inhibits the growth of the human lung adenocarcinoma A-549 in nude mice Cancer Res 2001 61 4 1280 1284 11245420

[B50] Eymin B Haugg M Droin N Sordet O Dimanche-Boitrel MT Solary E p27Kip1 induces drug resistance by preventing apoptosis upstream of cytochrome c release and procaspase-3 activation in leukemic cells Oncogene 1999 18 7 1411 1418 10.1038/sj.onc.1202437 10050878

[B51] Dimanche-Boitrel MT Micheau O France D Hammann A Duchamp O Genne P Solary E P27KiP1 overexpression inhibits the growth and doxorubicin sensitivity of HT29 human colon cancer cells in vivo Anticancer Res 2000 20 2A 849 852 10810365

[B52] Masuda A Osada H Yatabe Y Kozaki K Tatematsu Y Takahashi T Hida T Takahashi T Takahashi T Protective function of p27(KIP1) against apoptosis in small cell lung cancer cells in unfavorable microenvironments Am J Pathol 2001 158 1 87 96 10.1016/S0002-9440(10)63947-8 11141482 PMC1850277

[B53] Denicourt C Dowdy SF Cip/Kip proteins: more than just CDKs inhibitors Genes Dev 2004 18 8 851 855 10.1101/gad.1205304 15107401

[B54] Taniguchi T Endo H Chikatsu N Uchimaru K Asano S Fujita T Nakahata T Motokura T Expression of p21(Cip1/Waf1/Sdi1) and p27(Kip1) cyclin-dependent kinase inhibitors during human hematopoiesis Blood 1999 93 12 4167 4178 10361114

